# Cytoprotective Role of Autophagy in CDIP1 Expression-Induced Apoptosis in MCF-7 Breast Cancer Cells

**DOI:** 10.3390/ijms25126520

**Published:** 2024-06-13

**Authors:** Ryuta Inukai, Kanako Mori, Masatoshi Maki, Terunao Takahara, Hideki Shibata

**Affiliations:** Department of Applied Biosciences, Graduate School of Bioagricultural Sciences, Nagoya University, Furo-cho, Chikusa-ku, Nagoya 464-8601, Japan; ryootlytenten0309@gmail.com (R.I.); kanako.mkhn8@gmail.com (K.M.); mmaki@agr.nagoya-u.ac.jp (M.M.); takahara@agr.nagoya-u.ac.jp (T.T.)

**Keywords:** apoptosis, autophagy, CDIP1, proapoptotic protein

## Abstract

Cell death-inducing p53-target protein 1 (CDIP1) is a proapoptotic protein that is normally expressed at low levels and is upregulated by genotoxic and endoplasmic reticulum stresses. CDIP1 has been reported to be localized to endosomes and to interact with several proteins, including B-cell receptor-associated protein 31 (BAP31) and apoptosis-linked gene 2 (ALG-2). However, the cellular and molecular mechanisms underlying CDIP1 expression-induced apoptosis remain unclear. In this study, we first demonstrated that CDIP1 was upregulated after treatment with the anticancer drug adriamycin in human breast cancer MCF-7 cells but was degraded rapidly in the lysosomal pathway. We also demonstrated that treatment with the cyclin-dependent kinase 5 (CDK5) inhibitor roscovitine led to an increase in the electrophoretic mobility of CDIP1. In addition, a phosphomimetic mutation at Ser-32 in CDIP1 resulted in an increase in CDIP1 expression-induced apoptosis. We also found that CDIP1 expression led to the induction of autophagy prior to apoptosis. Treatment of cells expressing CDIP1 with SAR405, an inhibitor of the class III phosphatidylinositol 3-kinase VPS34, caused a reduction in autophagy and promoted apoptosis. Therefore, autophagy is thought to be a defense mechanism against CDIP1 expression-induced apoptosis.

## 1. Introduction

Macroautophagy (hereafter referred to as autophagy) is the major form of autophagy in which cytoplasmic macromolecules and organelles are engulfed by double-membrane structures, termed autophagosomes, and then delivered to the lysosome for degradation and recycling. In normal cells, autophagy occurs at a low level to sustain cellular homeostasis and prevent the accumulation of damaged organelles and aberrant proteins. Autophagy can also be upregulated under different stress conditions, such as nutrient starvation, hypoxia, oxidative stresses, and infections, as an adaptative response. Autophagy dysfunction has been implicated in the pathogenesis of numerous human diseases, including cancer as well as metabolic diseases, neurodegenerative diseases, and inflammatory and autoimmune diseases [1,2]. In cancer, autophagy plays dual roles in different stages and contexts of tumor development [3,4]. During the early stage of tumorigenesis, autophagy acts as a tumor-suppressive mechanism by suppressing oxidative stress and genomic instability. Conversely, in established or advanced tumors, many cancers exhibit a high autophagic flux that supports their survival, growth, and malignancy. Hence, manipulation of autophagy has been a promising and effective strategy in anticancer therapy [5,6,7]. Understanding the molecular mechanisms underlying the involvement of autophagy in anticancer drug-induced apoptosis of cancer cells is becoming increasingly important for improving combination chemotherapy.

Cell death-inducing p53-target protein 1 (CDIP1; also known as lipopolysaccharide-induced tumor necrosis factor-α factor-like, or LITAFL) is a proapoptotic protein that induces apoptosis in cells treated with the anticancer drug etoposide [8]. In cells with an intact p53 allele, CDIP1 is upregulated by activated p53 in response to genotoxic stresses induced by DNA-damaging agents. Overexpression of CDIP1 then leads to upregulation of TNF-α and increased sensitivity to TNF-α-induced cell death [8,9]. CDIP1 is also upregulated following treatment of cells with endoplasmic reticulum (ER) stress inducers: brefeldin A, tunicamycin, thapsigargin, and 15-deoxy, Δ^12,14^-prostamide J_2_ [10,11]. Knockdown studies have shown that upregulated CDIP1 is critical for apoptosis after genotoxic and ER stresses [8,10]. Under ER stress conditions, CDIP1 interacts with B-cell receptor-associated protein 31 (BAP31), followed by cleavage of BAP31 and promotion of the association of BAP31 with BCL-2 to induce the mitochondrial apoptosis pathway [10]. In cells expressing CDIP1, apoptosis-linked gene 2 (ALG-2), a proapoptotic Ca^2+^-binding protein, enhances the association between CDIP1 and tumor susceptibility gene 101 (TSG101) to promote apoptosis [12]. CDIP1 and its paralog lipopolysaccharide-induced tumor necrosis factor-α factor homolog (LITAF; also known as small integral membrane protein of the late endosome, SIMPLE, and p53-induced gene 7, PIG7) are a novel class of monotopic membrane proteins that embed into the cytosolic face of the membrane of endosomal compartments via their conserved C-terminal region [13,14]. CDIP1 has been shown to localize to CD63-positive late endosomes and LAMP1-positive endo/lysosomal compartments [13]. In addition, a function of CDIP1 and LITAF/SIMPLE in the membrane scission of tubular recycling endosomes has recently been pointed out [15]. However, the relationship between the endo/lysosomal localization of CDIP1 and apoptosis remains unclear.

In this study, we demonstrated that CDIP1 is a substrate protein for degradation by the lysosome. We also found that autophagy and apoptosis were induced by the expression of CDIP1 in a sequence in which autophagy preceded apoptosis. CDIP1 expression-induced apoptosis was enhanced with increased concentrations of a specific inhibitor of VPS34, SAR405, that prevents autophagy, suggesting a protective role of autophagy in CDIP1 expression-induced apoptosis. 

## 2. Results

### 2.1. Lysosomal Degradation of CDIP1

**Figure 1:**
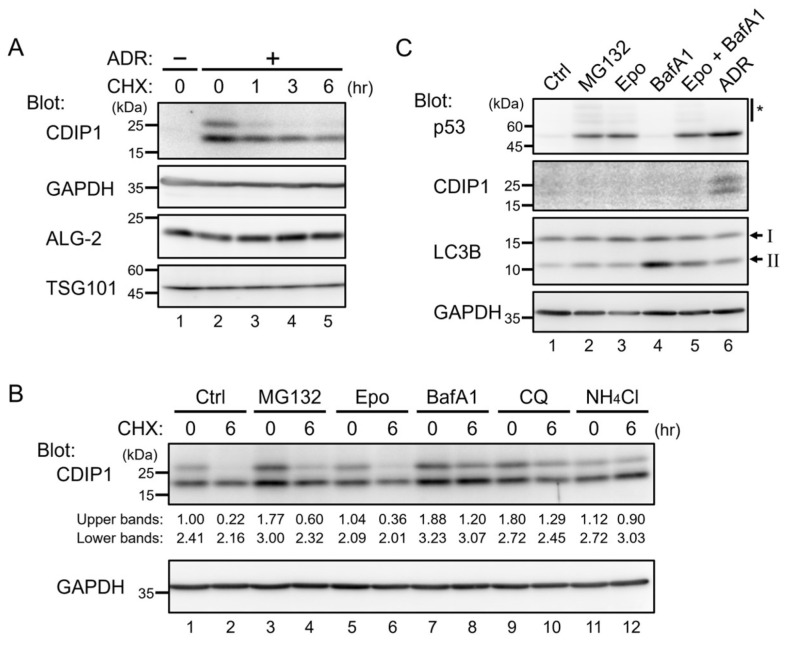
Cell death−inducing p53-target protein 1 (CDIP1) is upregulated by adriamycin and degraded via the lysosomal pathway in MCF-7 cells. (**A**) MCF-7 cells were cultured in a medium with or without 1 μM adriamycin (ADR) for 15 h (lanes 1 and 2), and then 50 μg/mL cycloheximide (CHX) was added for the indicated time periods (lanes 3–5). Cell lysates were analyzed by Western blotting with the indicated antibodies. (**B**) MCF-7 cells were cultured in a medium with 1 μM ADR for 24 h (lane 1), and then 50 μg/mL CHX was added for 6 h (lane 2). Proteasome and lysosome inhibitors as indicated (2 μM MG132, 2 μM epoxomicin [Epo], 10 nM bafilomycin A1 [BafA1], 100 μM chloroquine [CQ], and 50 mM NH_4_Cl) were added 1 h before the addition of CHX (lanes 3–12). Cell lysates were analyzed by Western blotting with the indicated antibodies. Intensities of the upper and lower bands of CDIP1 were quantified and normalized to the upper band of CDIP1 in lane 1. (**C**) MCF-7 cells were cultured in a medium with or without the indicated reagents for 6 h, and then cell lysates were analyzed by Western blotting with the indicated antibodies. The positions of LC3B-I and LC3B-II are indicated by I and II. Asterisk, slower−migrating ubiquitinated proteins.

CDIP1 belongs to a family of monotopic membrane proteins called LITAF family proteins with a highly conserved C-terminal region known as the LITAF domain or SIMPLE-like domain [13]. LITAF has been reported to be a short-lived protein with a half-life of about 2 h [16], which led us to examine whether CDIP1 is similarly turned over at a high rate in cells. As shown in Figure 1A, CDIP1 was present at barely detectable levels in MCF-7 human breast cancer cells under normal culture conditions (lane 1), while it was expressed as two bands at higher levels following administration of the anticancer drug adriamycin (ADR) (lane 2). This is consistent with the results of previous studies showing that CDIP1 is a target of p53 and can be expressed by DNA damage in a p53-dependent manner [8,9]. After induction of CDIP1 expression in MCF-7 cells by ADR, cycloheximide (CHX) was added to block further protein synthesis, and cultures were incubated for an additional 1, 3, or 6 h (Figure 1A, lanes 3–5). In the CHX chase assay, the protein level of CDIP1 gradually decreased with time after CHX treatment, and more than half was degraded in 6 h. In contrast, no change was observed for GAPDH, ALG-2, and TSG101. These results suggest that CDIP is a short-lived protein in MCF-7 cells treated with ADR. Next, to determine the degradation pathway of CDIP1, ADR-treated cells were cultured in a medium containing different inhibitors of degradation pathways for 1 h and then co-cultured with CHX for an additional 6 h. Treatment with MG132, a commonly used proteasome inhibitor, resulted in a slight decrease in the degradation of CDIP1 (Figure 1B, lanes 3 and 4). This seems to be a nonproteasome effect of MG132, possibly inhibition of cathepsin B, a lysosomal cysteine cathepsin [17], since treatment with epoxomicin (Epo), a more selective proteasome inhibitor [18], had no effect on degradation of CDIP1 (Figure 1B, lanes 5 and 6). Inhibition of proteasome activity by MG132 and epoxomicin was confirmed with an increase in p53 levels in MCF-7 cells after their exposure for 6 h (Figure 1C, lanes 2 and 3). We then assessed whether lysosomal inhibitors could affect the degradation of CDIP1. The lysosomotropic drugs bafilomycin A1 (BafA1), chloroquine (CQ), and ammonium chloride (NH_4_Cl) reduce the acidification of endocytic systems through different mechanisms. BafA1 inhibits V-ATPase, which pumps protons in the endo/lysosomal compartments [19]. CQ is accumulated in lysosomes and raises intra-lysosomal pH, while NH_4_Cl neutralizes intra-lysosomal pH at a much higher concentration than does CQ [20,21]. Compared to proteasome inhibitors, treatment of cells with BafA1 and NH_4_Cl resulted in more inhibition of the degradation of CDIP1 (Figure 1B, lanes 7, 8, 11, and 12). Treatment with CQ had similar effects but required higher concentrations. These results indicate that CDIP1 is degraded via the lysosomal pathway. In addition, CDIP1 was not detected in cells treated with BafA1 or BafA1 plus Epo (Figure 1C, lanes 4 and 5). Therefore, CDIP1 protein appeared not to be synthesized in cells under normal culture conditions. This is not the case for p53, which is usually maintained at a low level by a balance between protein synthesis and degradation by the proteasome.

**Figure 2:**
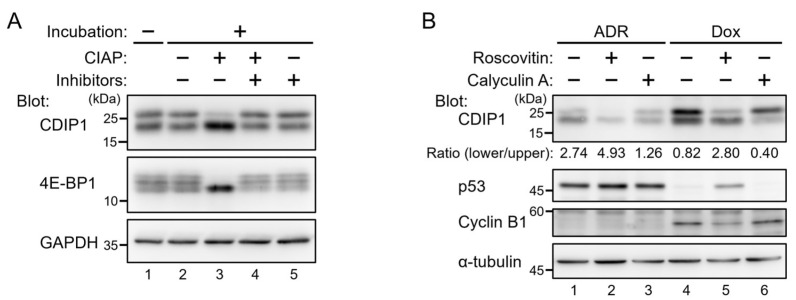
Effects of phosphatase treatment and incubation of cells with kinase and phosphatase inhibitors on the electrophoretic mobilities of CDIP1. (**A**) The cell lysate from MCF-7 cells expressing tetracycline−inducible CDIP1 (MCF-7 Tet-On CDIP1 cells) cultured in a medium with 100 ng/mL doxycycline for 24 h was divided into five aliquots, which were incubated with calf intestine alkaline phosphatase (CIAP) and its inhibitors (50 mM NaF, 10 mM β-glycerophosphate, and 1 mM Na_3_VO_4_) either separately or in combination, and then analyzed by Western blotting with the indicated antibodies. (**B**) MCF-7 Tet-On CDIP1 cells were cultured in a medium with 1 μM ADR (lanes 1–3) or 10 ng/mL Dox (lanes 4–6) for 24 h. Fifty nM roscovitine and 50 nM calyculin A were added 24 h and 45 min before harvest, respectively. Cell lysates were analyzed by Western blotting with the indicated antibodies. Intensities of the upper and lower bands of CDIP1 were quantified, and the ratio of the lower band to the upper band was calculated.

CDIP1 in MCF-7 cells treated with ADR was detected as two bands of different mobility, of which the slower one appeared to decrease faster in the CHX chase assay (Figure 1A). Similar bands have been detected by the same antibody in an established MCF-7 cell line expressing tetracycline-inducible CDIP1 (MCF-7 Tet-On CDIP1 cells) (Figure 2A, lane 1) as well as in HEK293 cells treated with the ER stress inducer brefeldin A [12]. To determine the cause of the CDIP1 doublet, we incubated an aliquot of the cleared cell lysate from MCF-7 cells inducibly expressing CDIP1 with calf intestine alkaline phosphatase (CIAP) to determine whether dephosphorylation alters the mobility of the CDIP1 bands (Figure 2A, lane 3). 4E-BP1 is a known phosphoprotein and was, therefore, a useful marker in this experiment. As with no incubation, mock incubation did not alter the electrophoretic properties of the CDIP1 doublet (Figure 2A, lanes 1 and 2), while incubation with CIAP caused a disappearance of the upper band and an increased intensity of the lower band (Figure 2A, lane 3). In addition, incubation with CIAP in the presence of CIAP inhibitors resulted in no alteration in the CDIP1 doublet (Figure 2A, lane 4), confirming that the disappearance of the upper band was due to the phosphatase activity of CIAP and not to any proteases. These results indicate that the upper band of the CDIP1 doublet represents CDIP1 in phosphorylated form. To further examine whether CDIP1 became phosphorylated in cells, calyculin A, an inhibitor of type 1 and type 2A protein phosphatases [22,23], was applied extracellularly to the ADR-treated MCF-7 cells before preparing cell lysates. Western blot analysis showed that the ratio of the lower band to the upper band decreased (Figure 2B, lane 3). In contrast, treatment of MCF-7 cells expressing CDIP1 with an inhibitor of CDK5, roscovitine [24], resulted in an increase in the ratio of the lower band to the upper band of CDIP1 (Figure 2B, lane 2). These results suggest that CDIP1 is a phosphoprotein in cells and that the phosphorylation level of CDIP1 is determined by the balance between kinase and phosphatase activities. The reason for using roscovitine as a protein kinase inhibitor in this experiment is that CDK5 is the enzyme potentially responsible for the phosphorylation of Ser-32 in CDIP1, as described below.

**Figure 3:**
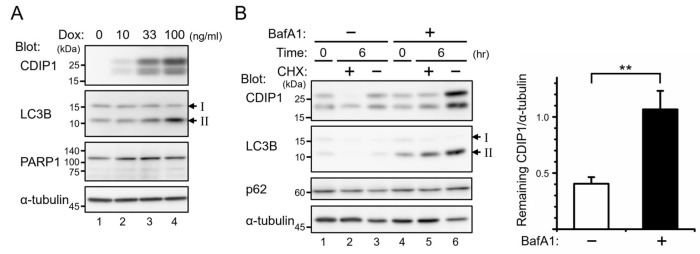
CDIP1 expressed in MCF-7 Tet-On CDIP1 cells is degraded via the lysosomal pathway. (**A**) MCF-7 Tet-On CDIP1 cells were cultured in a medium with Dox at the indicated concentrations for 24 h. Cell lysates were analyzed by Western blotting with the indicated antibodies. (**B**) MCF-7 Tet-On CDIP1 cells were cultured in a medium with 10 ng/mL Dox for 24 h, and then 50 μg/mL cycloheximide (CHX) was added for 6 h. Ten nM BafA1 was added 1 h before the addition of CHX. Cell lysates were analyzed by Western blotting with the indicated antibodies. The intensity of the CDIP1 band was normalized to α-tubulin, and then the remaining CDIP1 6 h after treatment with CHX was expressed relative to Time = 0 controls in the right graph. Data represent the mean ± SEM (*n* = 3). ** *p* < 0.01. The positions of LC3B-I and LC3B-II are indicated by I and II.

To exclude the possibility that lysosomal degradation of CDIP1 occurs only in genotoxic stress conditions, we examined MCF-7 Tet-On CDIP1 cells. As shown in Figure 3A, treatment of cells with the tetracycline analog doxycycline (Dox) for 24 h led to a dose-dependent increase in CDIP1, which was detected as doublet bands. The upper band seemed to be a phosphorylated form since treatment of cells with calyculin A led to a shift of the lower band to the upper band (Figure 2B, lanes 4 and 6). Although CDIP1 is a potent inducer of apoptosis, the cleaved fragment of poly[ADP-ribose] polymerase 1 (PARP1), an indicator of apoptosis, was not detected 24 h after the administration of Dox (Figure 3A). Therefore, we examined the stability of Dox-induced CDIP1 in the CHX chase assay. The protein level of CDIP1 was reduced by 40% at 6 h after treatment with CHX (Figure 3B, left panel, lane 2, and right panel, BafA1 minus), which is similar to the reduction rate in the ADR-induced CDIP1 (Figure 1A). In contrast, a decrease in the protein level of CDIP1 by treatment with CHX was completely blocked by pretreatment with BafA1 (Figure 3B, left panel, lane 5, and right panel, BafA1 plus). In addition, treatment with BafA1 without CHX increased the protein level of CDIP1 (Figure 3B, left panel, lanes 3 and 6). These results indicate that CDIP1 is a short-lived protein under both genotoxic stress and non-stress conditions, and CDIP1 turnover is regulated by lysosomal degradation.

### 2.2. Induction of Autophagy by CDIP1

**Figure 4:**
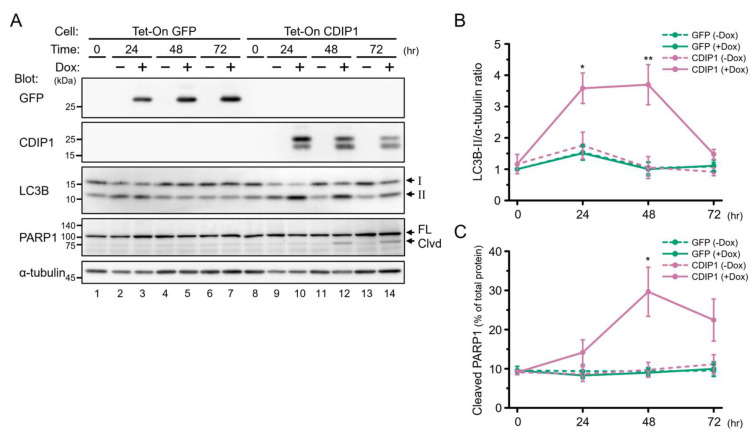
Expression of CDIP1 stimulates LC3B-II formation, preceding the production of cleaved PARP1 in MCF-7 cells. (**A**) MCF-7 Tet-On GFP cells and MCF-7 Tet-On CDIP1 cells were cultured in a medium with or without 100 ng/mL Dox for the indicated time periods. Cell lysates were analyzed by Western blotting with the indicated antibodies. The positions of LC3B-I and LC3B-II are indicated by I and II. The positions of full−length and cleaved PARP1 are indicated by FL and Clvd. (**B**) Intensities of the LC3B-II band were normalized to α-tubulin, and the LC3B-II/α-tubulin ratio was expressed relative to Time = 0. (**C**) Intensities of cleaved and full-length PARP1 bands were quantified, and then the percentage of cleaved PARP1 was determined as the ratio of the cleaved band intensity to the total intensity. Data represent means ± SEM (*n* = 3). * *p* < 0.05, ** *p* < 0.01.

During the course of the study on degradation of CDIP1, a good correlation was found between expression of CDIP1 and the amount of LC3B-II in MCF-7 Tet-On CDIP1 cells 24 h after administration of Dox (Figure 3A). LC3B is processed by ATG4-family cysteine proteases into LC3B-I immediately after its synthesis, and during autophagy, LC3B-I is conjugated to phosphatidylethanolamine to become LC3B-II, which is recruited to autophagosomal membranes and degraded by autophagy [25]. Therefore, the progression of autophagy can be monitored by the conversion of LC3B-I to LC3B-II and the degradation of LC3B-II. To exclude the possibility that the increased amount of LC3B-II is caused by culturing cells with Dox rather than by expression of CDIP1, we established a control cell line inducibly expressing GFP (MCF-7 Tet-On GFP cells) and examined the effects of treatment with Dox on the amount of LC3B-II. As shown in Figure 4A, lanes 1–7, and Figure 4B, expression of GFP in the MCF-7 Tet-On GFP cells after administration of Dox showed no effect on the amount of LC3B-II. Therefore, expression of CDIP1, rather than treatment with Dox, in MCF-7 Tet-On CDIP1 cells causes an increased amount of LC3B-II. Next, to determine when apoptosis was initiated in MCF-7 Tet-On CDIP1 cells following treatment with Dox, a time-course study was performed. The cleaved fragment of PARP1 was significantly increased in MCF-7 Tet-On CDIP1 cells 48 and 72 h after administration of Dox (Figure 4A, lanes 12 and 14, and Figure 4C). In contrast, the amount of LC3B-II in MCF-7 Tet-On CDIP1 cells reached a maximum between 24 and 48 h and then decreased, reaching levels similar to those in Dox-nontreated cells at 72 h (Figure 4A, lanes 8, 10, 12, and 14, and Figure 4B).

**Figure 5:**
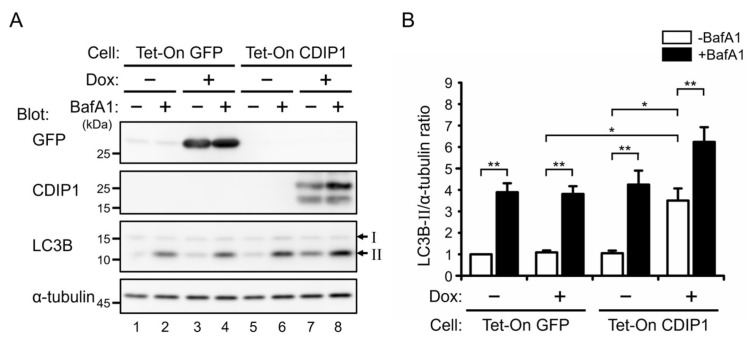
Expression of CDIP1 promotes autophagic flux in MCF-7 cells. (**A**) MCF-7 Tet-On GFP cells and MCF-7 Tet-On CDIP1 cells were cultured in a medium with or without 100 ng/mL Dox for 24 h. Ten nM bafilomycin A1 (BafA1) was added 1 h before harvest. Cell lysates were analyzed by Western blotting with the indicated antibodies. The positions of LC3B-I and LC3B-II are indicated by I and II. (**B**) Intensities of the LC3B-II band were normalized to α-tubulin, and the LC3B-II/α-tubulin ratio was expressed relative to the lysate of MCF-7 Tet-On GFP cells cultured in a medium without Dox and BafA1. Data represent means ± SEM (*n* = 4). * *p* < 0.05, ** *p* < 0.01.

To examine whether the increase in the amount of LC3B-II in MCF-7 Tet-On CDIP1 cells was due to induction of autophagy or impaired lysosomal degradation of LC3B-II, an autophagy flux assay was performed in the absence and presence of BafA1 as previously described [26]. Treatment of MCF-7 Tet-On GFP cells with BafA1 resulted in a significant increase in LC3B-II levels in both Dox-treated and nontreated conditions with similar signal intensities (Figure 5A,B, lanes 1–4). This indicates that treatment of cells with Dox has no effect on basal autophagic flux under normal cell culture conditions. In MCF-7 Tet-On CDIP1 cells, the amount of LC3B-II was increased in the Dox-treated condition (Figure 5A, lanes 5 and 7), and the amount was further increased upon incubation with BafA1 (Figure 5A, lanes 7 and 8, and Figure 5B). These results suggest that the expression of CDIP1 promotes autophagic flux in MCF-7 cells.

**Figure 6:**
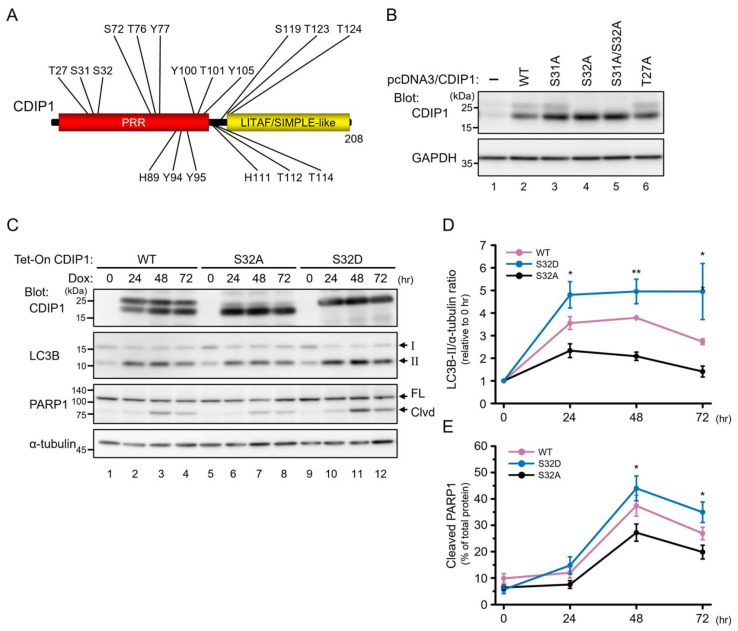
Effects of amino acid substitution of Ser at position 32 in CDIP1 with Ala or Asp on autophagy and apoptosis. (**A**) Schematic depiction of CDIP1 with positions of Ala substitutions labeled. PRR, pro−rich region. (**B**) HEK293 cells were transfected with expression plasmids for CDIP1 (WT) or mutants (S31A, S32A, S31A/S32A, or T27A). Mock-transfected cells (−) were used as a reference to identify bands corresponding to overexpressed proteins. Cell lysates were analyzed by Western blotting with the indicated antibodies. (**C**) MCF-7 Tet-On CDIP1 cells (WT), MCF-7 Tet-On CDIP1 S32A cells (S32A), and MCF-7 Tet-On CDIP1 S32D cells (S32D) were cultured in a medium with 100 ng/mL Dox for the indicated time periods. Cell lysates were analyzed by Western blotting with the indicated antibodies. The positions of LC3B-I and LC3B-II are indicated by I and II. The positions of full−length and cleaved PARP1 are indicated by FL and Clvd. (**D**) Intensities of the LC3B-II band were normalized to α−tubulin, and the LC3B-II/α-tubulin ratio was expressed relative to Time = 0. (**E**) Intensities of cleaved and full−length PARP1 bands were quantified, and then the percentage of cleaved PARP1 was determined as the ratio of the cleaved band intensity to the total intensity. Data represent means ± SEM (*n* = 3). * *p* < 0.05, ** *p* < 0.01.

Next, to determine which of the two bands is responsible for the autophagic and proapoptotic activities of CDIP1, we investigated the phosphorylation sites causing the band shift of CDIP1. We first predicted the phosphorylation sites of CDIP1 using the PhosphoSitePlus database (https://www.phosphosite.org/ date last accessed on 3 May 2024), which provides comprehensive information on post-translational modification sites, including phosphorylation sites, mainly derived from reported data from both low-throughput and high-throughput analyses such as mass spectrometry [27]. We found in the database that only one amino acid residue, Thr-27, of the N-terminus of human CDIP1 has been reported as a phosphorylation site [28] (Figure 6A). We exogenously expressed CDIP1 and its mutants in HEK293 cells and performed WB analysis. The endogenous level of CDIP1 in HEK293 cells was too low to be reliably detectable by the commercially available antibody against CDIP1 (Figure 6B, lane 1). In contrast, the Western blot of cells exogenously expressing CDIP1 produced two clear bands corresponding to CDIP1 (Figure 6B, lane 2). The band shift of the T27A mutant having alanine instead of Thr-27 was indistinguishable from that of the wild-type protein (Figure 6B, lanes 2 and 6). These findings do not exclude Thr-27 as a phosphorylation site but suggest that the presence of Thr-27 is not responsible for the band shift of CDIP1. We then focused on other residues in the N-terminal region because the doublet band of CDIP1 was observed in cells expressing the deleted mutant with the N-terminal region. Mutants of CDIP1 having alanine substitutions in serine, threonine, tyrosine, and histidine at the N-terminus (Figure 6A) were expressed in HEK293 cells and analyzed by Western blot. Among the mutants, the S32A and S31A/S32A substitutions caused the disappearance of the slower mobility band (Figure 6B, lanes 4 and 5). This suggests that the presence of Ser-32 is critical for the band shift of CDIP1. This residue is also predicted by the NetPhos 3.1 website server (https://services.healthtech.dtu.dk/service.php?NetPhos-3.1 date last accessed on 3 May 2024) as one of the potential phosphorylation sites of cyclin-dependent kinase 5 (cdk5, NetPhos score 0.589). Hence, in Figure 2B, we tested the effect of the CDK5 inhibitor roscovitine (Figure 2B, lanes 2 and 5). To examine the effect of a mutation of Ser-32 either to alanine or to aspartate to mimic phosphorylation on the autophagic and proapoptotic activities of CDIP1, we established MCF-7 cells inducibly expressing CDIP1 S32A and CDIP1 S32D. As shown in Figure 6C, lanes 6–8, the expressed CDIP1 S32A mutant proteins showed mobility similar to the faster mobility band of wild-type CDIP1, with some faint bands of slower mobility. In contrast, the expressed CDIP1 S32D mutant proteins showed mobility similar to the slower mobility band of wild-type CDIP1 (Figure 6C, lanes 10–12). These findings support the importance of Ser-32 in the band shift of CDIP1. A time-course study of the effects of the expression of the CDIP1 mutants revealed an early (24 h) 2-fold elevation of LC3B-II and a late (48 h) 1.6-fold increase in the cleaved ratio of PARP1 in cells expressing the S32D mutant compared to those in cells expressing the S32A mutant (Figure 6C–E). At 72 h, the cleaved ratio of PARP1 was reduced in the three cell lines, which may be due to reduced CDIP1 expression. Thus, there is a possibility that the phosphorylation of CDIP1 at Ser-32 is not required for its autophagic and proapoptotic activities, but the phosphorylation may enhance both the autophagic and proapoptotic activities of CDIP1.

### 2.3. Protective Role of Autophagy in CDIP1 Expression-Induced Apoptosis

**Figure 7:**
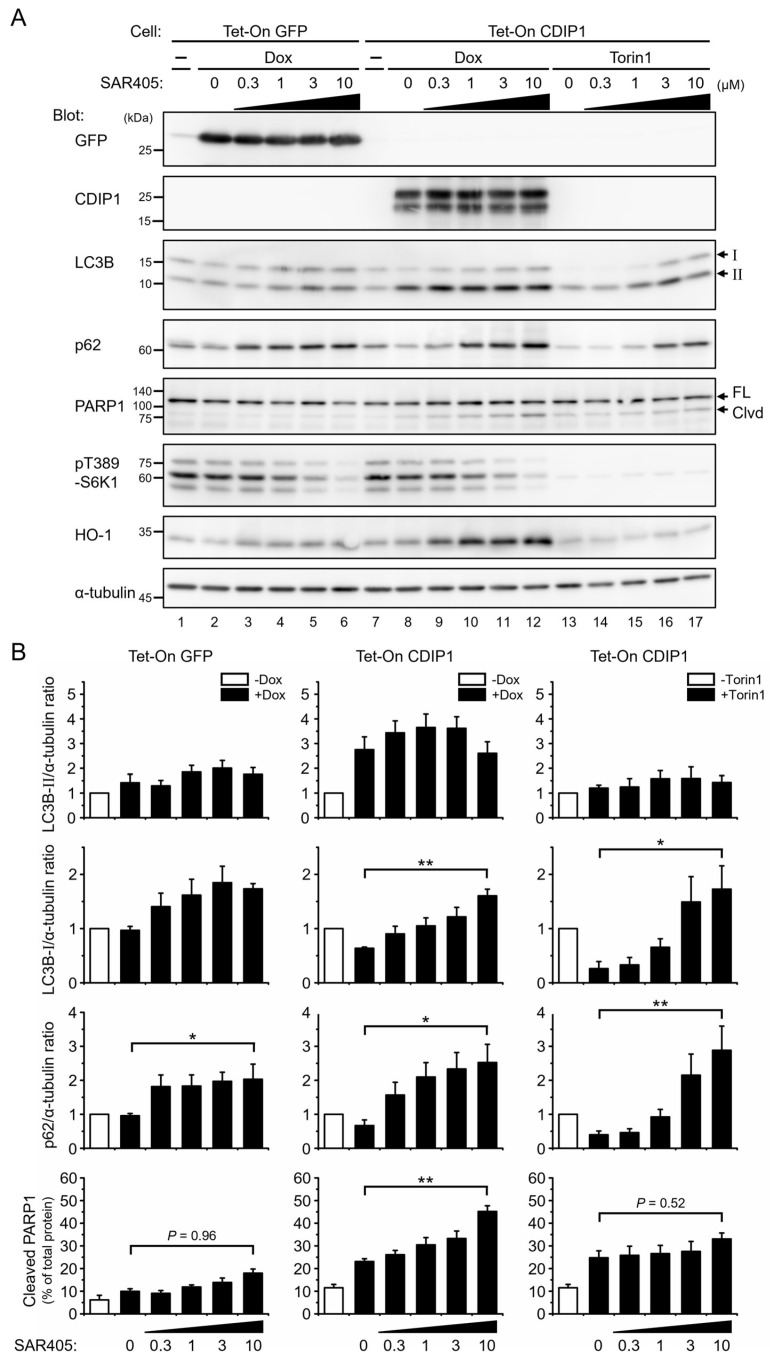
Effects of the PIK3C3/VPS34 inhibitor SAR405 on autophagy and apoptosis in MCF-7 cells inducibly expressing CDIP1. (**A**) MCF-7 Tet-On GFP cells and MCF-7 Tet-On CDIP1 cells were cultured in a medium with 100 ng/mL Dox or 250 nM Torin1 in combination with the indicated concentrations of SAR405 for 24 h. Cell lysates were analyzed by Western blotting with the indicated antibodies. The positions of LC3B-I and LC3B-II are indicated by I and II. The positions of full−length and cleaved PARP1 are indicated by FL and Clvd. (**B**) Intensities of the bands were quantified. LC3B−II/α−tubulin ratio (**top**), LC3B-I/α-tubulin ratio (**second**), and p62/α-tubulin ratio (**third**) were expressed relative to the cells cultured in a medium without Dox. The percentage of cleaved PARP1 (**bottom**) was determined as the ratio of the cleaved band intensity to the total intensity. Data represent means ± SEM (*n* = 3). * *p* < 0.05, ** *p* < 0.01.

Our time-course studies revealed that autophagy precedes the onset of apoptosis in MCF-7 cells after the expression of CDIP1. The next question was whether autophagy was required for the process of apoptosis in cells expressing CDIP1 or, in other words, whether CDIP1 expression-induced apoptosis was autophagic apoptosis or not. To test this, we applied the PIK3C3/VPS34 inhibitor SAR405 to prevent the formation of new autophagosomes during the induction of CDIP1 expression. PIK3C3/VPS34 is the catalytic subunit of the class III phosphatidylinositol 3-kinase (PtdIns3K) that plays critical roles in autophagosome generation through the formation of PtdIns3K complex I and in autophagosome maturation and endocytic trafficking through the formation of PtdIns3K complex II [29,30,31,32]. SAR405 is identified as a highly potent and selective inhibitor of PIK3C3/VPS34 in vitro and reduces autophagy by preventing the formation of autophagosomes in cells [33,34]. To evaluate the inhibition of autophagy, the protein levels of LC3B and p62 (also called sequestosome 1, SQSTM1) were examined [35]. p62/SQSTM1 is an autophagy receptor and is used as another marker for autophagy since it is degraded by autophagy [36,37]. When MCF-7 Tet-on GFP cells were treated with Dox and different concentrations of SAR405 for 24 h, there was little difference in the amounts of LC3B-II and LC3B-I, but the amount of p62 increased in a dose-dependent manner (Figure 7A, lanes 1–6). The latter finding suggests inhibition of the basal level of autophagy by SAR405 in cells expressing GFP. To examine the effect of SAR405 on the autophagy of MCF-7 Tet-on CDIP1 cells, we treated cells with SAR405 in combination with Torin1, an inhibitor of the mechanistic target of rapamycin (mTOR) that negatively regulates autophagy [38,39]. Inhibition of mTOR by Torin1 was monitored by Western blot, which showed a decrease in phosphorylation levels of its substrate, S6 kinase 1 (S6K1). As shown in Figure 7A, lanes 7 and 13, and Figure 7B, administration of Torin1 without SAR405 resulted in a slight increase in LC3B-II and a discernable decrease in LC3B-I. The amount of p62 was also decreased in cells treated with Torin1. These results indicate the promotion of autophagy flux by Torin1 in MCF-7 Tet-on CDIP1 cells. When cells were treated with Torin1 and various concentrations of SAR405 (Figure 7A, lanes 14–17), LC3B-I and p62 were increased in a concentration-dependent manner for SAR405. Therefore, Torin1-induced autophagy flux in this cell line is effectively inhibited by SAR405. In contrast, the amount of cleaved fragments of PARP1 was small and was not affected by the addition of SAR405 in this condition. We then treated cells with Dox instead of Torin1 to induce CDIP1 expression and autophagy in combination with SAR405. As shown in Figure 7A, lanes 7 and 8, treatment of cells with Dox for 24 h caused a marked increase in CDIP1 and LC3B-II and a decrease in LC3B-I and p62. A small amount of cleaved PARP1 fragments was also detected. Similar to the Torin1-induced autophagy, simultaneous administration of Dox and SAR405 resulted in a dose-dependent increase in the levels of LC3B-I and p62 for SAR405 (Figure 7A, lanes 8–12, and Figure 7B). Although the level of LC3B-II was not affected by the addition of SAR405, the increase in LC3B-I and p62 suggests that the autophagy flux induced by CDIP1 expression is inhibited by the addition of SAR405. In addition, a clear difference between cells treated with Torin1 and cells treated with Dox was observed in a Western blot using the antibody against PARP1; in cells treated with Dox and SAR405, increased intensity of cleaved fragments of PARP1 was detected in a concentration-dependent manner for SAR405. This indicates that inhibition of autophagy flux promotes the onset of apoptosis in cells expressing CDIP1, and, hence, CDIP1 expression-induced apoptosis is not a form of autophagic apoptosis; rather, autophagy has a protective role in CDIP1 expression-induced apoptosis. Treatment of CDIP1-expressing cells with SAR405 resulted in an increase in heme oxygenase-1 (HO-1), a sensitive indicator of cellular oxidative stress [40,41]. In contrast, SAR405 had no or little effect on HO-1 expression in GFP-expressing or Torin1-treated cells. Thus, it is possible that CDIP1 expression causes oxidative stress and that autophagy in cells expressing CDIP1 may play a role in preventing the accumulation of oxidants or scavenging them.

**Figure 8:**
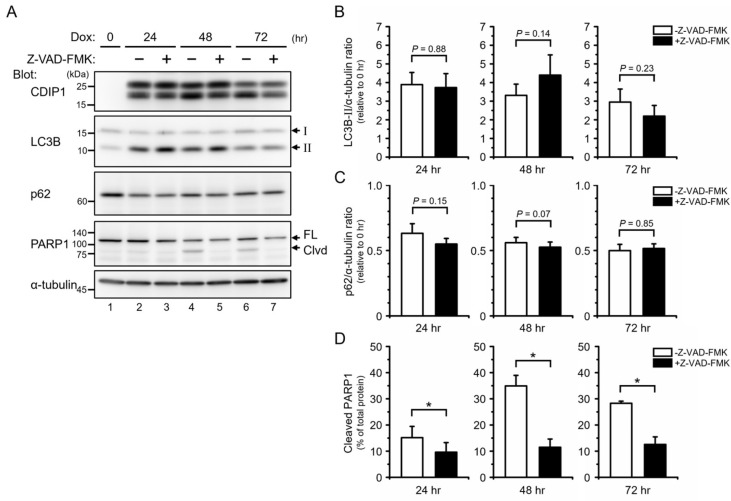
Effects of the caspase inhibitor Z−VAD−FMK on autophagy and apoptosis in MCF-7 cells inducibly expressing CDIP1. (**A**) MCF-7 Tet-On CDIP1 cells were cultured in a medium with 100 ng/mL Dox in combination with or without 10 μM Z−VAD−FMK for the indicated time periods. Cell lysates were analyzed by Western blotting with the indicated antibodies. The positions of LC3B-I and LC3B-II are indicated by I and II. The positions of full−length and cleaved PARP1 are indicated by FL and Clvd. (**B**) Intensities of the LC3B-II band were normalized to α−tubulin, and the LC3B-II/α-tubulin ratio was expressed relative to Time = 0. (**C**) Intensities of the p62 band were normalized to α-tubulin, and the p62/α-tubulin ratio was expressed relative to Time = 0. (**D**) Intensities of cleaved and full−length PARP1 bands were quantified, and then the percentage of cleaved PARP1 was determined as the ratio of the cleaved band intensity to the total intensity. Data represent means ± SEM (*n* = 3). * *p* < 0.05.

We next examined the effects of inhibition of caspases by benzyloxycarbonyl-Val-Ala-Asp (O-methyl) fluoromethylketone (Z-VAD-FMK) [42] on autophagy after induction of CDIP1 expression at three time points: 24, 48, and 72 h. Treatment with Z-VAD-FMK inhibited the cleavage of PARP1 at all time points tested but had no significant effect on the amount of LC3B-II and p62 (Figure 8). These results indicate that caspase activities are not required for CDIP1 expression-induced autophagy, and the results are consistent with the idea that autophagy precedes apoptosis after induction of CDIP1 expression.

## 3. Discussion

CDIP1 is a proapoptotic protein that triggers pathways leading to caspase activation. Accumulating evidence indicates that the expression of CDIP1 is precisely controlled at transcriptional and post-transcriptional levels in different cellular contexts. Transcription of human CDIP1 is upregulated by p53 in several types of cancer cells exposed to genotoxic stresses by anticancer agents [8]. Transcriptional activation of CDIP1 is also regulated by endoplasmic reticulum stresses, but it has been reported to be p53-independent, and the pathways remain unidentified [10]. In addition, multiple microRNAs modulate CDIP1 expression. Most of them have been shown to recognize their respective binding sites in the non-coding 3′ untranslated region of the CDIP1 gene [43,44,45,46,47]: downregulation of microRNA-214-3p in human neuroblastoma SH-SY5Y cells after stimulation with 1-methyl-4-phenylpyridinium ion (MPP+) promotes CDIP1 expression [46], while microRNA-21-5p in exosomes released from cardiac telocytes silences CDIP1 expression in endothelial cells that have received exosomes to inhibit apoptosis during myocardial infarction [45].

In this study, we demonstrated the post-translational properties of CDIP1. First, the cycloheximide chase assay revealed that CDIP1 is a short-lived protein in MCF-7 cells treated with ADR (Figure 1A) and in MCF-7 cells expressing tetracycline-inducible CDIP1 (Figure 3B). Although it is currently unclear whether CDIP1 proteins are degraded in a constitutive or regulated manner, it is likely that protein synthesis rates must exceed the degradation in order to maintain the expression level of CDIP1 in the cell, which activates transcription and downregulates miRNAs that target CDIP1 mRNA. Second, experiments on the degradation pathway using inhibitors showed that lysosomes, not proteasomes, are responsible for the degradation of CDIP1 (Figure 1B and Figure 3B). CDIP1 has been shown to be a monotopic membrane protein and to be localized to endosomal organelles via the C-terminal region [13]. In this context, the results of the present study showing lysosomal degradation of CDIP1 provide supporting evidence that CDIP1 is a membrane-integrated protein. On the other hand, for LITAF, a paralog of CDIP1, some reported data indicate that it is degraded mainly by lysosomes [16], while other data suggest that both the lysosomal and proteasomal pathways are involved in its degradation [48]. Differences in degradation pathways may reflect differences in the membrane-localized proportions of LITAF proteins or may result from differences in experimental conditions. Recently, LITAF and CDIP1 were identified as host receptors for the *Bacillus cereus* Hemolysin BL toxin [49]. In such cases, the C-termini of these proteins are exposed to the cell surface and appear to function as toxin receptors. The C-terminal region of the tail-anchored proteins is post-translationally inserted into the membrane [50]. There may be some regulatory mechanism that determines whether the C-termini of CDIP1 and LITAF are exposed to the cell surface as single-pass transmembrane proteins or are located in the cytosol as monotopic membrane proteins. It is also possible that CDIP1 and LITAF proteins with different C-terminal topologies may differ in their stability. Since the ubiquitination of CDIP1 at Lys-21, Lys-198, and Lys-205 is listed in the PhosphoSitePlus database, the ubiquitin system might be involved in the lysosomal degradation of CDIP1. Third, we found that CDIP1 undergoes phosphorylation with a band shift in SDS-PAGE. Mutant analyses demonstrated that Ser-32 is responsible for the band shift of CDIP1. Treatment with the CDK5 inhibitor roscovitine decreased the band intensity of slower migrating forms of CDIP1, raising the possibility that CDK5 is the protein kinase that is responsible for the phosphorylation of CDIP1 at Ser-32. CDK5 is a proline-directed kinase that preferentially phosphorylates a substrate with a canonical consensus motif, [S/T]PX[R/K/H]. The sequence from Ser-32 to the C-terminus of CDIP1, ^32^SPAV^35^, does not match this motif, while about half of the known substrates of CDK5 have been shown to be phosphorylated at sites that do not contain a canonical consensus motif [51]. It should also be noted that roscovitine is a potent inhibitor of CDK5 as well as other CDKs, including CDK1, CDK2, and CDK7, and a weak inhibitor of other kinases such as CaMK2, CK1α, CK1δ, DYRK1A, EPHB2, ERK1, ERK2, FAK, and IRAK4 [24,52]. As shown in Figure 2B, the upper band of CDIP1 is increased preferentially in MCF-7 Tet-On CDIP1 cells by Dox treatment. In ADR-treated cells, CDIP1 is predicted to be expressed after the cell cycle is arrested. Therefore, the low intensity of the upper band of CDIP1 in the ADR-treated cells may suggest that the kinase responsible for CDIP1 phosphorylation at Ser-32 is not active in cells with an arrested cell cycle. The identification of protein kinases that phosphorylate CDIP1 in cells remains an important issue for future analysis.

In the course of investigating the degradation pathway of CDIP1, accumulation of LC3-II was detected first in MCF-7 cells expressing CDIP1, followed by cleavage of PARP1, an indicator of caspase-3/7 activation (Figure 4). Yan et al. recently reported an accumulation of LC3-II in the colorectal cancer cell lines RKO and LoVo after overexpression of CDIP1 [47]. We performed an autophagy flux assay by treating MCF-7 cells expressing CDIP1 with the lysosome inhibitor bafilomycin A1, and we confirmed that the accumulation of LC3-II was due to autophagy promotion (Figure 5). Since autophagy flux was enhanced prior to apoptosis induced by CDIP1 expression, this apoptosis was considered autophagic cell death, but treatment with the autophagy inhibitor SAR405 did not block apoptosis but rather accelerated apoptosis induction (Figure 7). Therefore, autophagy in CDIP1-expressing cells may have a potential physiological protective role against apoptosis. CDIP1 is abundantly expressed in most of the cancer cell lines with wild-type p53 [9], but these cell lines are resistant to apoptosis despite high levels of CDIP1 expression. Although it is unclear whether most cancer cells that express large amounts of CDIP1 have high autophagy flux, previous studies using autophagy-deficient mice have shown that pancreatic tumor development driven by oncogenic Ras requires autophagy in the presence of wild-type p53 [53]. The findings in this study provide useful insight into the role of autophagy in cells with CDIP1 expression. If cancer cells express high levels of CDIP1 and have high autophagy flux, it is conceivable that the administration of anti-autophagy drugs could be effective in cancer therapy to block tumor development.

## 4. Materials and Methods

### 4.1. Antibodies and Reagents

The following commercially available antibodies were used for Western blot analysis: rabbit polyclonal antibodies against CDIP1 (13824, Cell Signaling Technology, Danvers, MA, USA), C16orf5 (CDIP1) (PAB16708, Abnova, Taipei, Taiwan), LC3B (3868, Cell Signaling Technology, Danvers, MA, USA), PARP1 (9542, Cell Signaling Technology, Danvers, MA, USA), HO-1/HMOX1 (10701-1-AP, Proteintech, Rosemont, IL, USA); rabbit monoclonal antibodies against 4EBP1 (clone 53H11, 9644, Cell Signaling Technology, Danvers, MA, USA), phosphor-p70 S6 kinase (Thr389) (clone 108D2, 9234, Cell Signaling Technology, Danvers, MA, USA); mouse monoclonal antibodies against GAPDH (clone 6C5, sc-32233, Santa Cruz Biotechnology, Santa Cruz, CA, USA), TSG101 (clone 4A10, GTX70255, GeneTex, Irvine, CA, USA), p53 (clone DO-1, sc-126, Santa Cruz Biotechnology, Santa Cruz, CA, USA), CCNB1/cyclin B1 (clone GNS1, sc-245, Santa Cruz Biotechnology, Santa Cruz, CA, USA), α-tubulin (clone DM1A, T9026, Sigma-Aldrich, Saint Louis, MO, USA), p62 (clone 3/P62 LCK LIGAND, 610833, BD Biosciences, Franklin Lakes, NJ, USA), GFP (clone B2, sc-9996, Santa Cruz Biotechnology, Santa Cruz, CA, USA). Rabbit polyclonal antibodies against ALG-2 were prepared as described previously [54]. Goat antibodies against mouse IgG and rabbit IgG conjugated with horseradish peroxidase (HRP) were from Jackson ImmunoResearch (West Grove, PA, USA). Adriamycin (040-21521), MG132 (135-18453), Epoxomicin (336-43811), Bafilomycin A1 (029-11643), Chloroquine Diphosphate (036-17972), Ammonium Chloride (017-02995), and Calyculin A (038-14453) were purchased from FUJIFILM Wako Pure Chemical Corporation (Osaka, Japan). Blasticidin S (ant-bl-1) and Puromycin (ant-pr-1) were from InvivoGen (San Diego, CA, USA). Cycloheximide was from Nacalai Tesque (06741-91, Kyoto, Japan). Bafilomycin A1 was from AdipoGen Corp. (BVT-0252, San Diego, CA, USA). Roscovitine was from Santa Cruz Biotechnology (sc-24002, Santa Cruz, CA, USA). SAR405 was from ChemScene LLC (CS-5756, Monmouth Junction, NJ, USA). Torin1 was from LKT Laboratories (T5870, St. Paul, MN, USA). Z-Val-Ala-Asp(Ome)-CH_2_F (Z-VAD-FMK) was from Peptide Institute (3188-v, Osaka, Japan). Alkaline Phosphatase (Calf intestine) was from Takara Bio (2250A, Kusatsu, Shiga, Japan).

### 4.2. Plasmid Constructions

Expression plasmids for CDIP1 mutants were constructed by site-directed mutagenesis using pcDNA3/CDIP1 [12] as a template and each pair of primers (Table 1). pLenti CMVTRE3G eGFP Puro (w819-1) was a gift from Dr. Eric Campeau (Addgene plasmid #27570). The DNA fragments encoding CDIP1 S32A and CDIP1 S32D were amplified from pcDNA3/CDIP1 S32A and pcDNA3/CDIP1 S32D, respectively, with a pair of primers: forward 5′-gtaccgcgggcccgggatccgcgaagatgtccagcgagcctcc-3′ and reverse 5′-acaagaaagctgggtctagattagcacaggcgcttgtacg-3′. The products were inserted between the BamHI and XbaI sites of pLenti CMVTRE3G eGFP Puro using a seamless ligation cloning extract (SLiCE) [55]. The resultant plasmids were designated pLenti CMVTRE3G CDIP1 S32A Puro and pLenti CMVTRE3G CDIP1 S32D Puro. All sequences of the constructed plasmids were verified by DNA sequencing.

### 4.3. Cell Culture, DNA Transfection and Lentiviral Infection

HEK293 cells, HEK293T cells, and MCF-7 cells were cultured in DMEM (Nissui, Tokyo, Japan) supplemented with 5% (for HEK293 cells and HEK293T cells) or 10% (for MCF-7 cells) fetal bovine serum (FBS), 4 mM _L_-glutamine, 100 U/mL penicillin G, and 100 μg/mL streptomycin at 37 °C under humidified air containing 5% CO_2_. For transient expression of CDIP1 and its mutants, one day after HEK293 cells were seeded and cultured, cells were transfected with the expression plasmid DNAs by using the conventional PEI-MAX method (24764, Polysciences, Warrington, PA, USA). For establishing Tet-On system-induced MCF-7 cells, MCF-7 cells stably expressing rtTA3 [12] were infected with lentivirus that was produced from HEK293T cells transiently transfected with respective lentivectors pLenti CMVTRE3G eGFP Puro, pLenti CMVTRE3G CDIP1 S32A Puro, or pLenti CMVTRE3G CDIP1 S32D Puro in combination with packing plasmid psPAX2 and envelop plasmid pMD2.G by using FuGENE6 (Promega, Madison, WI, USA) as previously described [12]. pMD2.G (Addgene plasmid #12259) and psPAX2 (Addgene plasmid #12260) were gifts from Dr. Didier Trono. The established cell lines were designated MCF-7 Tet-On GFP, MCF-7 Tet-On CDIP1 S32A, and MCF-7 Tet-On S32D, respectively, and cultured in DMEM supplemented with 10% FBS, 4 mM _L_-glutamine, 100 U/mL penicillin, 100 μg/mL streptomycin, 1 μg/mL puromycin, and 10 μg/mL blasticidin S at 37 °C under humidified air containing 5% CO_2_.

### 4.4. Cell Lysate Preparation and Western Blotting

Cells were washed with ice-cold phosphate-buffered saline (PBS) (137 mM NaCl, 2.7 mM KCl, 8 mM Na_2_HPO_4_, and 1.5 mM KH_2_PO_4_, pH 7.4) and lysed with lysis buffer A (62.5 mM Tris-HCl, pH 6.8, 2% SDS, 10% glycerol) containing protease inhibitors (0.3 mM pefabloc, 9 μg/mL leupeptin, 3 μM E-64, 3 μM pepstatin A, and 0.6 mM phenylmethylsulfonyl fluoride) and phosphatase inhibitors (50 mM NaF, 10 mM β-glycerophosphate, and 1 mM Na_3_VO_4_). After sonication followed by centrifugation at 10,000× *g* for 10 min at 4 °C, the protein concentrations of the obtained cleared cell lysates were adjusted by measuring protein concentration using the BCA Protein Assay Kit (ThermoFisher Scientific, Waltham, MA, USA). After the addition of 2-mercaptoethanol and bromophenol blue at final concentrations of 5% and 0.002%, respectively, followed by boiling for 5 min, equal amounts of total protein lysates were analyzed by Western blotting with specific antibodies.

In Figure 2A, MCF-7 Tet-On CDIP1 cells were cultured in a medium containing 100 ng/mL Dox for 24 h and harvested with PBS. Cells were then lysed with lysis buffer B (20 mM HEPES-KOH, pH 7.2, 142.5 mM KCl, 1.5 mM MgCl_2_) containing 0.1% Triton X-100 and protease inhibitors (0.3 mM pefabloc, 9 μg/mL leupeptin, 3 μM E-64, 3 μM pepstatin A, and 0.6 mM phenylmethylsulfonyl fluoride). After centrifugation, cleared cell lysates were mixed with an equal volume of buffer C (100 mM Tris-HCl, pH 8.5, 0.2 mM Zn(OAc)_2_, 2 mM MgCl_2_) and incubated with 28 units of calf intestine alkaline phosphatase (CIAP) for 24 h in the presence or absence of phosphatase inhibitors (50 mM NaF, 10 mM β-glycerophosphate, and 1 mM Na_3_VO_4_). The samples, untreated with CIAP, were also prepared. Protein samples were resolved by SDS-PAGE, followed by Western blotting with specific antibodies.

### 4.5. Statistical Analysis

Statistical analysis was performed by one-way analysis of variance (ANOVA), followed by Tukey’s test using Origin 9.1 (Micro Software, Northampton, MA, USA). *p*-values less than 0.05 are considered statistically significant.

### 4.6. Research Ethics

We followed biosafety guidelines for recombinant DNA research at Nagoya University. Experimental proposals were approved by the Recombinant DNA Biosafety Committee of the Graduate School of Bioagricultural Sciences, Nagoya University: Nou15-067 (approved on 24 March 2006) and Nou19-002 (approved on 12 April 2019).

## Figures and Tables

**Table 1:** Oligonucleotide primers used for site-directed mutagenesis of CDIP1.

Mutations	Sence Primer Sequences	Antisense Primer Sequences
T27A	5′-agccccgcccgccccaggccgtt-3′	5′-aacggcctggggcgggcggggct-3′
S31A	5′-cccaggccgtgcctccccagc-3′	5′-gctggggaggcacggcctggg-3′
S32A	5′-caggccgttccgccccagctgtg-3′	5′-cacagctggggcggaacggcctg-3′
S32D	5′-caggccgttccgacccagctgtg-3′	5′-cacagctgggtcggaaccgcctg-3′
S31A/S32A	5′-cccaggccgtgccgccccagctgtg-3′	5′-cacagctggggcggcacggcctggg-3′
S72A/T76A/Y77A	5′-caccacacatggctgcagatggcgccgccatgcctccg-3′	5′-cggaggcatggcggcgccatctcagccatgtgtggtg-3′
H89A	5′-ctccaggccccgccccacccatg-3′	5′-catgggtggggcggggcctggag-3′
Y94A/Y95A	5′-cacccatgggcgccgcccccccaggg-3′	5′-ccctgggggggcggcgcccatgggtg-3′
Y100A/Y105A	5′-ccccagggcccgccacgccagggcccgcccctggccctg-3′	5′-cagggccaggggcgggccctggcgtggcgggccctgggg-3′
H111A	5′-cctgggggcgccacagccacag-3′	5′-ctgtggctgtggcgcccccagg-3′
T112A/T114A	5′-ctgggggccacgcagccgcagtcctggtc-3′	5′-gaccaggactgcggctgcgtggcccccag-3′
S119A	5′-gtcctggtccctgcaggagctgccac-3′	5′-gtggcagctcctgcagggaccaggac-3′
T123A/T124A/T126A	5′-caggagctgccgccgcagtggcagtgctgcag-3′	5′-ctgcagcactgccactgcggcggcaggtcctg-3′

## Data Availability

The original contributions presented in the study are included in the article, further inquiries can be directed to the corresponding author.

## Abbreviations

ADR	adriamycin
ALG-2	apoptosis-linked gene 2
BafA1	bafilomycin A1
BAP31	B-cell receptor-associated protein 31
CDIP1	cell death-inducing p53-target protein 1
CHX	cycloheximide
CIAP	calf intestine alkaline phosphatase
CQ	chloroquine
Dox	doxycycline
Epo	epoxomicin
ER	endoplasmic reticulum
GAPDH	glyceraldehyde-3-phosphate dehydrogenase
LC3B	microtubule-associated proteins 1A/1B light chain 3B
LITAF	lipopolysaccharide-induced tumor necrosis factor-α factor homolog
PARP1	poly[ADP-ribose] polymerase 1
SIMPLE	small integral membrane protein of the late endosome
TNF-α	tumor necrosis factor-α
TSG101	tumor susceptibility gene 101
